# Disrupting Motor Cortical Regional Activity during Motor Sequence Skill Training Impairs Human Motor Visuomotor Skill Acquisition and Learning That Is Not Sequence-Specific

**DOI:** 10.1523/ENEURO.0348-25.2026

**Published:** 2026-05-22

**Authors:** Iran Gutierrez, Oindrila Sinha, Nathan Baune, Jasmine Mirdamadi, Michael Borich

**Affiliations:** ^1^Department of Rehabilitation Medicine, Emory University, Atlanta, Georgia 30322; ^2^School of Biological Sciences, Georgia Institute of Technology, Atlanta, Georgia 30332; ^3^Department of Biomedical Engineering, Georgia Institute of Technology, Atlanta, Georgia 30332

**Keywords:** implicit motor learning, motor cortical regions, sequence motor learning, transcranial magnetic stimulation

## Abstract

Implicit sequence and visuomotor skill learning is important for successful goal-directed behavior in everyday tasks. However, prior research has primarily relied on correlational methods to investigate the underlying neural mechanisms of sequence and visuomotor skill learning. To evaluate the necessary contributions of different motor cortical regions to both types of skill learning, we enrolled 62 neurotypical adults (41 females, 21 males) and delivered spatiotemporally resolved single-pulse transcranial magnetic stimulation (TMS) over either the premotor cortex (PMC) or primary motor cortex (M1) to transiently disrupt activity while participants practiced an implicit motor sequence task. We hypothesized that (1) PMC disruption would preferentially reduce sequence-specific skill acquisition (Experiment 1) and retention (Experiment 2), while (2) M1 disruption would diminish visuomotor skill acquisition and retention but not sequence learning. Our results demonstrated that TMS-based interference over both M1 and PMC did not disrupt implicit sequence-specific motor skill learning after training; however, it did disrupt visuomotor skill acquisition and total learning that was not sequence-specific. Furthermore, disruption of PMC activity had a greater effect on reducing visuomotor skill learning than M1 supporting a potentially distinct role of the PMC in the early stages of skill learning.

## Significance Statement

Determining which brain areas are required for motor sequence learning is crucial to understanding goal-directed behaviors in everyday life. However, the causal contributions of regional brain activity to implicit sequence learning are poorly understood. Here we used single-pulse transcranial magnetic stimulation, a form of noninvasive brain stimulation, to interfere with activity in either premotor (PMC) or primary motor (M1) cortex during implicit motor sequence learning. Our results highlight that both regions are engaged during learning and that PMC may play a unique role in general visuomotor acquisition and may contribute to sequence-specific skill learning. Study findings could contribute to the identification of neural biomarkers necessary to develop precise and personalized neuromodulation strategies for enhancing of motor skill learning and/or recovery.

## Introduction

Whether typing a password on a phone, playing a chord on a guitar, or tying one's shoelaces, the acquisition of motor sequences is fundamental to goal-directed behavior and functional independence in daily life. These everyday actions may seem effortless, but each relies on motor learning processes supported by dynamic interactions between perceptual, cognitive, and motor systems that transform sensory input into purposeful, coordinated actions (Krakauer et al., 2019). With repeated training, skill acquisition and consolidation processes support improved sequence skill performance with control transitioning from deliberate, attention-dependent processing to more automatic execution (Fitts and Posner, n.d.).

The early acquisition phase of motor skill learning is characterized by activation of a distributed network, including cognitive control regions such as the dorsolateral prefrontal cortex, premotor cortex (PMC), supplementary motor area, and associative regions of the striatum, particularly the caudate nucleus (Honda et al., 1998; Doyon et al., 2003; Penhune and Steele, 2012 ). This early stage involves the encoding of new sequence information and transforming sensory input into task-relevant motor representations. As performance becomes more fluent with extensive training, neural activity shifts toward greater engagement of the primary motor cortex (M1) and sensorimotor striatum (e.g., putamen), reflecting a transition from cognitive and planning-related processes to more efficient, execution-level motor control (Doyon and Benali, 2005; Dayan and Cohen, 2011 ).

Within this framework, M1 has long been recognized for a key role in motor execution and in the consolidation of well-learned skills (Wilkinson et al., 2010; Yokoi et al., 2018). In contrast, the PMC has been consistently implicated in action planning (Churchland et al., 2006; Hoshi and Tanji, 2007), sensorimotor integration (Hoshi and Tanji, 2007; Falaki et al., 2024), and sequential motor control (Tanji and Shima, 1994; Hikosaka et al., 2002; Solopchuk et al., 2016). Functional imaging has revealed sustained activation in the right premotor cortex during motor sequence learning, particularly under conditions promoting explicit awareness of the sequence (Grafton et al., 1995), and animal studies have shown that PMC neurons fire in chunk-specific patterns, suggesting a role in structuring action sequences (Kurata and Tanji, 1986; Mushiake et al., 1991; Tanji and Shima, 1994; Hikosaka et al., 2002; Perlman et al., 2010).

Despite converging evidence, the causal contribution of PMC to implicit sequence skill learning remains unresolved. Prior literature frequently relied on correlational methods such as fMRI or conflated implicit and explicit processes due to task designs that permit awareness of the sequence (Bischoff-Grethe et al., 2004). Neuromodulation approaches, such as transcranial magnetic stimulation (TMS) or transcranial direct current stimulation, can causally probe the contributions of specific brain regions during different phases of learning (Pascual-Leone et al., 1994; Grafman and Wassermann, 1998; Cantarero et al., 2013). For example, PMC or M1 stimulation applied before, during, or after sequence training can alter different aspects of online acquisition versus offline consolidation processes (Kantak and Winstein, 2012; Meehan et al., 2013). However, traditional neuromodulation protocols have variable after-effects over a broad timespan ranging from minutes to hours and thus blur the distinction between planning and execution of individual movements. Therefore, we still lack a mechanistic account of exactly when and to what extent during the course of skill training that each region is necessary for information encoding that results in both sequence-specific and general visuomotor skill learning.

To test the necessary contributions of PMC and M1 to implicit motor sequence acquisition and general visuomotor skill learning, the present study employed neuro-navigated, single-pulse TMS to interfere with local cortical activity while practicing a modified version of the serial reaction time task (SRTT; Nissen and Bullemer, 1987). In implicit versions of the SRTT, participants show improvement in performance without conscious awareness of an embedded sequence, isolating procedural learning from declarative knowledge (Willingham, 1998; Fu et al., 2008). Subthreshold TMS time-locked to the premovement phase was delivered either over right PMC, right M1, or in a sham configuration to interfere with cortical activity preceding individuated finger movements during training of a repeating 12-item sequence. We tested two parallel hypotheses: first, that PMC stimulation would selectively diminish sequence-specific acquisition and retention compared with M1 or sham TMS, reflecting its key role in action planning and sequence encoding; second, that M1 stimulation would reduce visuomotor skill compared with PMC and sham TMS across both phases that would not be sequence-specific, consistent with M1's role in execution and visuomotor skill consolidation. To test these hypotheses, we designed an implicit SRTT protocol incorporating skill assessments before and after a single training exposure followed by a retention assessment in a subset of participants to test the distinct contributions of PMC and M1 to the acquisition, retention, and overall learning of both sequence-specific and visuomotor skill in a cohort of neurotypical young adults.

## Materials and Methods

### Experimental design

**Figure 1.**
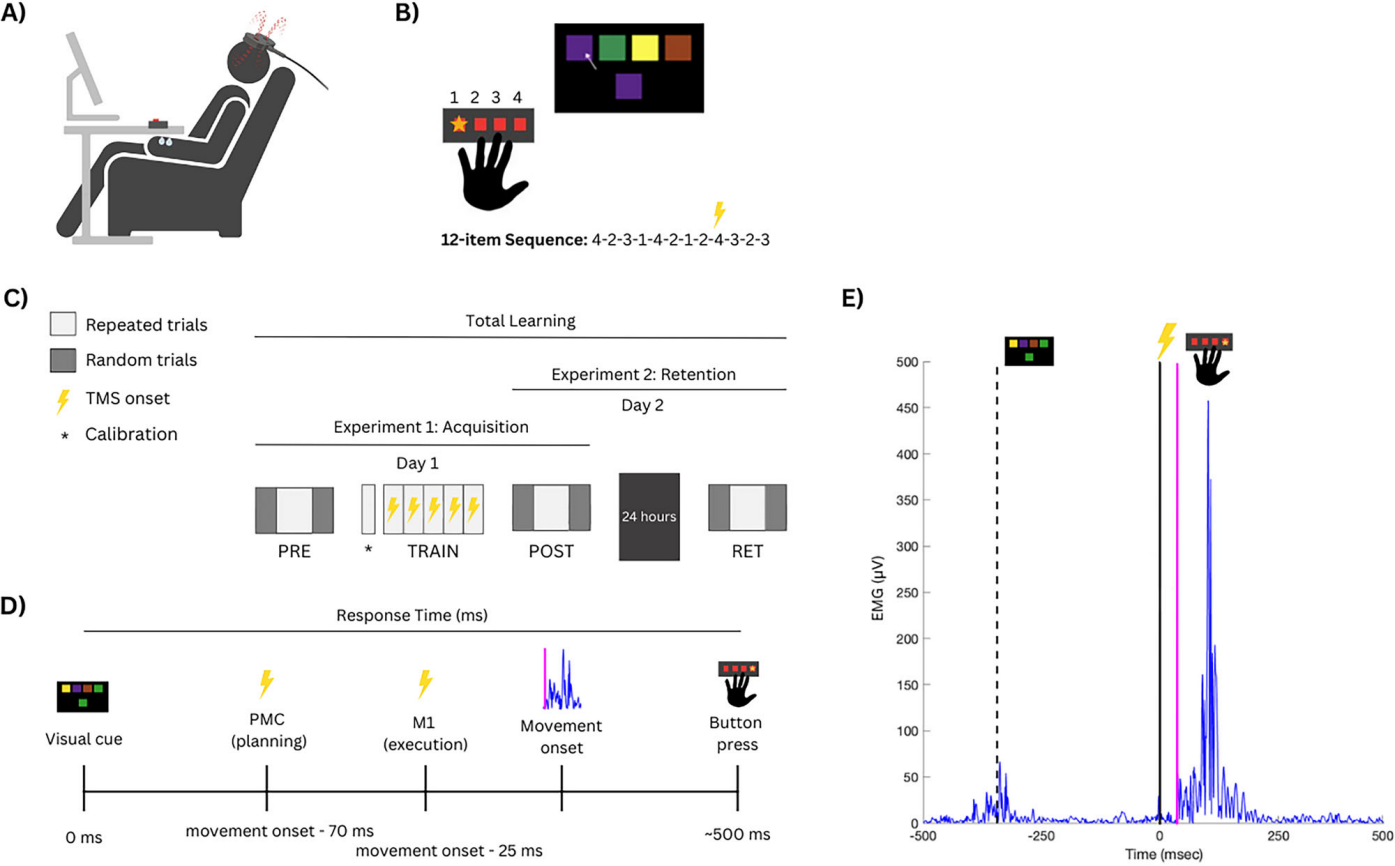
Overall experiment design. ***A***, Experimental setup. ***B***, Participants were instructed to press the button matching the color of the target square (e.g., leftmost target—Digit 5 of the left hand). A 12-item sequence was embedded (numbered to demonstrate order of digit presses; buttons or targets are not visually numbered here to demonstrate the order of the digit presses oriented from left to right; this information was not provided to the participant), and then TMS was delivered to the representation of left FDI in right M1 once per sequence repeat during the training blocks. ***C***, All participants completed Day 1 (TRAIN, PRE, and POST test), and a subset of participants completed retention testing 24 h later (Day 2, RET test) to assess within-session skill acquisition (Experiment 1) and skill learning (Experiment 2) following a single training session. Prior to training, a calibration block (*) for task familiarization was performed and to determine initial timing of TMS delivery for the first training block. ***D***, After visual cue onset (0 ms), subthreshold TMS (90% AMT) was delivered prior to movement onset at latencies targeting movement-related activity in PMC or M1. ***E***, Representative EMG data from a participant in the M1 TMS group showing TMS delivery prior to onset of cue-related EMG activity (solid magenta line) in the left FDI (visual cue presented at ∼−300 ms, dashed line).

Two experiments were conducted to investigate the functional contribution of PMC and M1 to implicit sequence-specific motor skill acquisition, retention, and total learning. In Experiment 1, all participants (*N* = 62; average age, 24.7; SD, 3 years; 20 males) completed an implicit motor sequence learning task and received active or sham TMS during training to assess neural mechanisms of implicit sequence-specific motor skill acquisition. Participants were randomized to the M1 (*N* = 19), PMC (*N* = 18), or Control (sham TMS; *N* = 20) group. In Experiment 2, the Control group and a subset of participants in each of the active stimulation groups returned 24 h (±4 h) later to assess motor skill retention and total learning (Fig. 1*C*). The distribution of participants per stimulation group for Experiment 2 was M1, *N* = 11; PMC, *N* = 12; and Control, *N* = 20.

### Participants

Human subjects were recruited at Emory University and tested at the Emory Rehabilitation Hospital. Eligibility criteria included right-handed dominance with no history of movement impairments, neurologic or psychiatric condition, contraindications to magnetic resonance imaging (MRI), or TMS. All participants gave written consent prior to participating in the study and the experimental protocol was approved by Emory University's Institutional Review Board. Sixty-two participants were initially enrolled. Five participants were excluded from the final data analyses due to TMS delivery occurring after movement onset (*n* = 2) and technical issues with EMG recordings to accurately determine TMS delivery timing (*n* = 3).

### Motor sequence learning task paradigm

A modified version of the SRTT (Nissen and Bullemer, 1987; Robertson, 2007) was used to evaluate both implicit sequence-specific and general visuomotor skill performance and learning using the same task due to its direct comparison between repeated (i.e., sequence-specific motor learning) and random (i.e., nonordered, sequenced motor learning) trials. Participants were seated comfortably with a monitor at the eye level and completed the task using their nondominant (left) hand on a custom-built four-button response box to reduce potential for ceiling effects (Fig. 1*B*). On each trial, a target square appeared below a row of four colored squares displayed on a computer monitor. Each color (purple, green, yellow, or brown) corresponded to one of four buttons, aligned with the participant's left fifth to second digits (left to right). Participants were instructed to press the button that matched the color of the target square as quickly and accurately as possible. Correct responses triggered a new trial, with all colors shifting to conceal the presence of a repeating sequence (Clos et al., 2018). At the start of each assessment and training block, participants were asked to read and follow standardized task instructions presented on the screen. Prior to the start of each block, participants had two practice trials to become familiarized with the task.

### SRTT tests and training protocol

All participants completed a behavioral protocol consisting of a pretraining test block (PRE), five training blocks, and one post-training test block (POST) for Experiment 1 as shown in Figure 1*C*. Each test block consisted of a total of 280 button presses (50 random, 180 repeated, 50 random). Random trial sections were uniquely generated for each participant and for each random section. Repeated trial sections consisted of 15 repeats of a 12-item sequence, and the same sequence was used across training and test blocks. The PRE test quantified baseline performance, while POST test assessed within-session changes in performance. There were a total of five training blocks, and each training block consisted of only repeated trials (15 instances of the same 12-item sequence). Experiment 2 involved an additional test block performed ∼24 h after completing the first experiment (RET). This test block was identical to the other test blocks consisting of repeated and random trials to evaluate the degree of skill retention and total learning.

Following completion of the study, participants were asked if they used any strategies to complete the task or if they noticed any details about the task to probe their awareness of a sequence. No participants endorsed awareness of a repeating pattern suggesting successful masking of the sequence.

### Noninvasive brain stimulation (TMS) to interfere with cortical activity

TMS was performed using a figure-of-eight coil (Magstim D70^2^) connected to a monophasic stimulator (Magstim M200^2^). Stimulation target identification and individualized thresholding were determined using real-time neuronavigation (Brainsight, Rogue Research) and electromyography (EMG; BrainAmp ExG, Brain Products).

#### Motor thresholding procedure

Active motor threshold (AMT) of the M1 corresponding to the left first dorsal interosseous (FDI) muscle was obtained for each participant using EMG to measure TMS-motor evoked potentials (defined as peak-to-peak amplitude of at least 200 µV), while participants isometrically abducted the FDI 20–25% of their maximum voluntary contraction using continuous visual biofeedback. An adaptive algorithm, ML-PEST (Awiszus, 2003), was used to determine the AMT.

#### TMS target sites: M1 and PMC

Participants were randomly assigned to receive TMS over either the right M1 or PMC during training. Standardized 3-D coordinates based on a meta-analysis of peak functional MRI motor task-related activity in the dorsal PMC (*X* = 30; *Y* = −4; *Z* = 58; Mayka et al., 2006) were used to identify the target location for each participant using anatomical MRI scans collected for participants normalized into Montreal Neurological Institute coordinate space. Participants in the control group did not undergo an anatomical MRI scan but the same targeting procedures were used with half the control group randomized to receive sham TMS over the standardized PMC target coordinates and the other half to receive sham TMS over M1. Real-time stereotactic navigation was used throughout the session to guide and ensure accurate TMS targeting during training.

#### TMS interference protocol

The TMS-based interference protocol during training consisted of a single TMS pulse delivered at 90% AMT over the assigned target site per sequence repeat (total of 15 stimulations per training block) immediately preceding a button press performed by the index finger. A subthreshold intensity was chosen to modulate brain activity without generating overt motor responses. During sham TMS delivery in the Control condition, the coil was positioned 90° orthogonal to the scalp to minimize any biological effects of the stimulation while maintaining auditory and somatosensory stimuli associated with TMS delivery. Timing of stimulation was calculated by taking the minimum response time (i.e., fastest) from the prior training block and subtracting 70 ms for PMC targeting and 25 ms for M1 targeting to time stimulation delivery toward the later motor planning and premotor execution phases, respectively, but prior to movement onset (Fig. 1*D*; Weinrich and Wise, 1982; Starr et al., 1988; Chen et al., 1998; Hussain et al., 2022). This conservative choice of TMS timing was to ensure stimulation occurred during the premovement stage irrespective of stimulation site and given the duration of TMS effects can last several hundred milliseconds (Farzan et al., 2013; Xiao et al., 2025). Response times were quantified as the time from visual onset to time of correct button press.

### Data analysis

#### Timing of TMS delivery

EMG data for each TMS trial were reviewed to confirm that TMS delivery occurred prior to the onset of the corresponding key press with the following group averages calculated: M1 (132 ± 71 ms), PMC (211 ± 39 ms), and Control (218 ± 184 ms; Table 2). Timing of TMS delivery relative to EMG onset was calculated and averaged per block. Individuals with TMS delivery occurring after EMG onset in over 50% of trials and those without confirmed individualized timing of TMS delivery were removed from the final analysis.

#### SRTT skill learning assessments

**Sequence-specific skill.** Following reviewer recommendation, the difference between mean response times for random and repeated trials was calculated to provide a sensitive measure of sequence-specific skill, with higher scores indicating greater implicit sequence-specific skill (Nissen and Bullemer, 1987; Willingham et al., 1989; Willingham and Goedert, 1999). Specifically, we calculated a skill score (SS) from the last 50 random trials and last four sequence repetitions in each test block (Willingham and Goedert, 1999; Robertson, 2007).

**General visuomotor skill.** Mean random response times (50 random prior and 50 random following repeated trials) and mean repeated response times (15 12-item repetitions) were extracted separately within each test block.

In Experiment 1, SS and individual response times from each trial type were calculated for the pre- and post-training test blocks to quantify implicit sequence-specific and general visuomotor skill acquisition. In Experiment 2, SS and individual response times from each trial type were calculated for the retention test block and compared with the pre- and post-training blocks to assess total skill learning and skill retention, respectively.

In addition to assessing response time as a measure of performance, the percentage of correct responses was also calculated for both trial types within each test block for all participants.

### Statistical analyses

Statistical analyses were conducted in R Studio (version 4.3.3; R Core Team, 2024) using the following functions/packages: emmeans (marginal means and contrasts; version 1.10.1; R. Lenth, 2023a), lmer (fit linear mixed-effects models; version 1.1-35.2; Bates et al., 2015), and lmerTest (*p* values; version 3.1-3; Kuznetsova et al., 2017). Data were analyzed using mixed-effects models, which provide several advantages for the current experimental design (Table 1). Mixed-effects models appropriately handle the hierarchical structure of our data (repeated measures nested within participants) while accommodating missing data points and unbalanced designs without requiring listwise deletion (Baayen et al., 2008; Barr et al., 2013). This approach is particularly well suited for response time data, which often exhibit non-normal distributions and heteroscedasticity that mixed-effects models can better accommodate compared with traditional ANOVA approaches. In conjunction, upon data inspection, three participants in the Control had substantially longer response times during the pretraining test block so primary analyses were re-run after removal of these participants to determine if group effects were driven, in part, by these data points.

**Table 1.** Statistical analyses

Analysis	Data structure	Statistical test	Power analysis
TMS timing validation	Response time outliers (IQR method); TMS timing validation relative to EMG onset	Response time outliers (IQR method); TMS timing validation relative to EMG onset	N/A
Experiment 1: Motor Skill Acquisition	Repeated-measures SS (normal distribution assumed); within-subject factors, Test (PRE, POST); between-subject factor, Group (Control, M1, PMC); *N* = 57 total (Control, *n* = 20; M1, *n* = 19; PMC, *n* = 18)	Linear mixed-effects model with participant random intercepts; post hoc pairwise comparisons using estimated marginal means with Kenward–Roger approximation	Effect sizes and 95% CIs reported in results; power analysis not conducted for complex mixed-effects design [see section X.Y (i.e., the stats analysis section)]
Experiment 2: retention and total learning	Repeated-measures SS (normal distribution assumed); within-subject factors, Test (PRE, POST, RET); between-subject factor, Group (Control, M1, PMC); *N* = 43 total (Control, *n* = 20; M1, *n* = 11; PMC, *n* = 12)	Linear mixed-effects model with participant random intercepts; post hoc pairwise comparisons using estimated marginal means with Kenward–Roger approximation	Effect sizes and 95% CIs reported in results; power analysis not conducted for complex mixed-effects design (see section X.Y)

Power analysis was not conducted a priori. Post hoc power calculations are not recommended for complex mixed-effects models with multiple factors and interactions, as they can be misleading and statistically inappropriate (Hoenig and Heisey, 2001). Instead, we report effect sizes with confidence intervals to facilitate interpretation and comparison with future studies.

An interquartile range (IQR) for response times (millisecond) was calculated within each test, training block, and individual. On average, all groups had 5% or less of test trials with response times outside of the IQR removed from the final analysis. Participants in the PMC and Control group had on average 3.5% of training trials removed while participants in the M1 groups had 5% of training trials removed. This approach aimed to minimize the influence of extreme trial values and ensure reasonable variability within each test and individual.

Given that stimulation condition contained three levels, traditional model summaries rely on dummy coding that produces pairwise comparisons against a reference category, making interpretation of the overall pattern of results less straightforward. To address this limitation and provide a more comprehensive understanding of experimental effects, estimated marginal means (emmeans) were employed for post hoc analyses and interpretation (R.V.Lenth, 2023b ). The emmeans approach calculates model-predicted means for each condition while controlling for other factors in the model, enabling direct comparison of all pairwise contrasts and facilitating interpretation of main effects and interactions across all three stimulation conditions.

### Experiment 1: acquisition phase analysis

To examine the differential contributions of PMC and M1 to implicit sequence-specific motor skill acquisition, a linear mixed-effects model for SS was constructed using the lmer function. The model included participants as random intercepts to account for individual differences in baseline performance. The fixed-effects structure comprised two main factors: Test (PRE, POST) and Group (PMC, M1, Control), along with possible two-way interactions. Control and PRE test conditions were set as reference categories to facilitate interpretation of learning-related changes. Model assumptions were verified through examination of residual plots and normality tests.

Post hoc pairwise comparisons were conducted using estimated marginal means with Kenward–Roger df approximation (Kenward and Roger, 1997) without adjustment for multiple comparisons, as our hypotheses specified directional predictions for each stimulation condition. The Kenward–Roger method provides more accurate Type 1 error control in mixed-effects models with small to moderate sample sizes by accounting for uncertainty in variance component estimation. This analytical approach allowed isolation of the specific effects of TMS disruption on sequence learning while controlling for general motor performance changes and individual variability.

To better understand potential effects of stimulation on practice-related changes in general motor skill development, an additional model was constructed using mean response time data for random versus repeated trials. The fixed-effects structure comprised of three main factors: Test (PRE, POST), Trial Type (Repeated, Random), and Group (PMC, M1, Control), along with all possible two-way and three-way interactions. Control, PRE, and Random conditions were set as reference categories to facilitate interpretation of learning-related changes.

### Experiment 2: retention phase and total learning analysis

To assess the role of PMC and M1 in implicit sequence-specific skill retention and total learning, an identical analytical framework to Experiment 1 was employed, with Test representing the comparison between (1) pretraining performance (PRE) and immediate post-training performance (POST), (2) immediate post-training performance (POST) and 24 h retention testing (RET), and (3) the comparison between pretraining performance (PRE) and 24 h retention testing (RET). This analysis was performed to assess the effects of TMS-based interference on offline consolidation and also on overall learning.

The fixed-effects structure included Test (PRE, POST, RET) and Group (PMC, M1, Control) with all interactions. Control and PRE conditions served as reference categories to highlight total learning-specific effects. Sequence-specific retention was quantified as the change in SS from POST to RET test blocks, with positive values indicating offline skill enhancement and negative values indicating offline forgetting of prior skill acquired. This approach distinguished between TMS effects on initial skill acquisition versus the offline processes supporting memory consolidation and long-term retention of implicit motor sequences.

An additional model was constructed to differentiate general motor skill versus sequence-specific skill using mean response time data for random versus repeated trials. The fixed-effects structure comprised of three main factors: Test (PRE, POST, RET), Trial Type (Sequence, Random), and Group (PMC, M1, Control), along with all possible two-way and three-way interactions. Control, PRE, and Random conditions were set as reference categories to facilitate interpretation of learning-related changes.

## Results

### TMS timing: TMS delivery occurs prior to movement onset

Across groups, an average of 8% of trials consisted of TMS delivered after EMG onset (M1, 12%; MC, 4%; Control, 7%). Five of 62 participants were removed from the final analyses for the following reasons: 2 of 62 (PMC, 2) individuals had over 50% of TMS delivered after EMG onset, 1 of 62 (PMC, 1) had technical issues with EMG during training preventing confirmation of TMS timing, and 2 of 62 (M1, 1; PMC, 1) did not have individualized TMS timings (Table 2).

**Table 2.** TMS delivery accuracy and exclusions

	M1	PMC	Control
Average TMS to EMG onset latency (millisecond)	132 ± 71	211 ± 39	218 ± 184
Average number of TMS trials occurring after EMG onset per participant (of 75 trials)	9 ± 8	3 ± 5	5 ± 7
Number of participants removed (62 enrolled)	1	4	0

Timing characteristics of TMS delivery by group, including mean TMS pulse latency from EMG onset (ms ± SD), mean number of late-delivery TMS trials per participant (mean ± SD), and participant exclusions due to TMS delivery timing postmovement onset (>50% of trials with TMS delivered after onset of EMG activity; *n* = 2; 1 M1 and 1 PMC). Due to technical issues with EMG recordings that prevented EMG onset determination, three participants in the PMC group were also excluded from the final analyses.

### Experiment 1: Disruption of both M1 and PMC activity during training reduce general visuomotor, but not sequence-specific, skill acquisition

**Figure 2.**
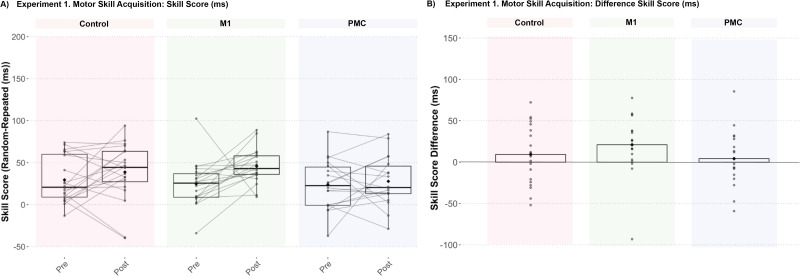
All groups, irrespective of active stimulation, demonstrated increased implicit sequence-specific motor skill acquisition from pre- to post-training (*p* < 0.05).

Across groups, there was a significant increase in SS from pre- to post-training (main effect of Test, estimate = −11.50 ms; SE = 4.69; *t* = −2.45; *p* = 0.017; 95% CI [−20.90, −2.10]) but was not specific to group (Test × Group interaction, all *p* > 0.1; Fig. 2). Baseline SS (PRE) was comparable across groups; however, following training (POST), SS increased in Control (mean ΔSS = −9.27 ms; SE = 7.91) and M1 (mean ΔSS = −21.01 ms; SE = 8.11) groups but showed the least amount of change in the PMC group (mean ΔSS = −4.21 ms; SE = 8.33).

**Figure 3.**
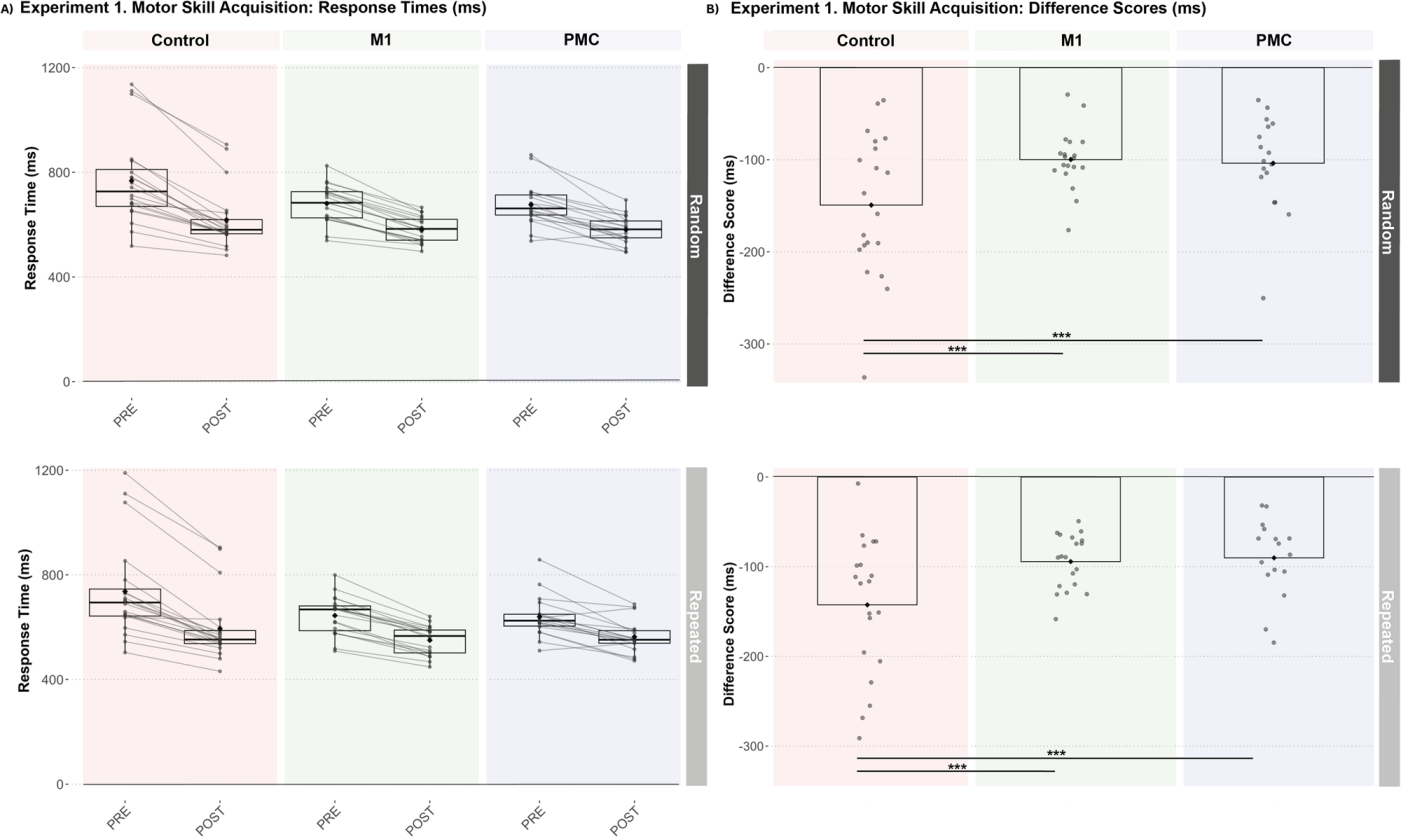
Stimulation groups demonstrated less within-session skill acquisition that was not sequence-specific. ***A***, All groups demonstrated decreased response times from pre- to post-training for both random and repeated segments within each test block (***p < 0.001). ***B***, However, less decrease in response times were shown in both stimulation groups compared with controls (***p < 0.001).

Irrespective of the group and test, individuals showed significantly faster response times on repeated sequence trials compared with random trials (Main Effect of Trial Type, estimate = 29.19 ms; SE = 4.79; *t* = 6.10; *p* < 0.001; 95% CI [19.73, 38.64]) but was not specific to test (Trial Type × Test interaction, estimate = 10.70 ms; SE = 9.57; *t* = 1.12; *p* = 0.27; 95% CI [−8.20, 29.61]) nor group (Trial Type × Group interaction, all *p* > 0.5). However, the control group showed a significantly greater reduction in response times across repeated and random trials from pre- to post-training compared with both active stimulation groups (Group × Test interaction, M1 estimate = 48.90 ms; SE = 11.56; *t* = 4.22; *p* < 0.001; 95% CI [26.02, 71.69]; PMC estimate = 59.48 ms; SE = 11.73; *t* = 5.07; *p* < 0.001; 95% CI [36.32, 82.64]) with no significant differences between the M1 and PMC stimulation groups (Group × Test interaction, estimate = −10.62 ms; SE = 11.87; *t* = −0.89; *p* = 0.37; 95% CI [−34.07, 12.83]; Fig. 3). Upon reviewing individual data points, three participants in the control group showed prolonged response times during the pretraining test block. To assess the impact on these data points on the group differences, the three participants were removed from the model, and the significant reduction in response time following training in controls compared with the active stimulation groups remained (Group × Test interaction, M1 estimate = 30.00 ms; SE = 10.66; *t* = 2.81; *p* = 0.006; 95% CI [8.93, 51.07]; PMC estimate = 40.62 ms; SE = 10.80; *t* = 3.76; *p* < 0.001; 95% CI [19.28, 61.96]).

Additional models were run for Experiment 1 to assess the impact of removing excluded participants on group differences. Even with the inclusion of excluded participants, our results for Experiment 1 did not change. Across all groups, SS significantly increased from pre- to post-training (main effect of Test, estimate = −11.77; SE = 4.44; *t* = −2.65; *p* = 0.010; 95% CI [−20.66, −2.88]). Additionally, improved performance across trials (Sequence and Random) was greatest in Controls compared with stimulation groups (Group × Test interaction, M1 estimate = 51.77; SE = 11.11; *t* = 4.66; *p* < 0.001; 95% CI [29.84, 73.69]; PMC estimate = 63.64; SE = 10.86; *t* = 5.86; *p* < 0.001; 95% CI [42.21, 85.06]).

### Experiment 2: Disruption of both M1 and PMC activity during training reduces general visuomotor, but not sequence-specific, skill retention, and total learning with greater effects with PMC stimulation

**Figure 4.**
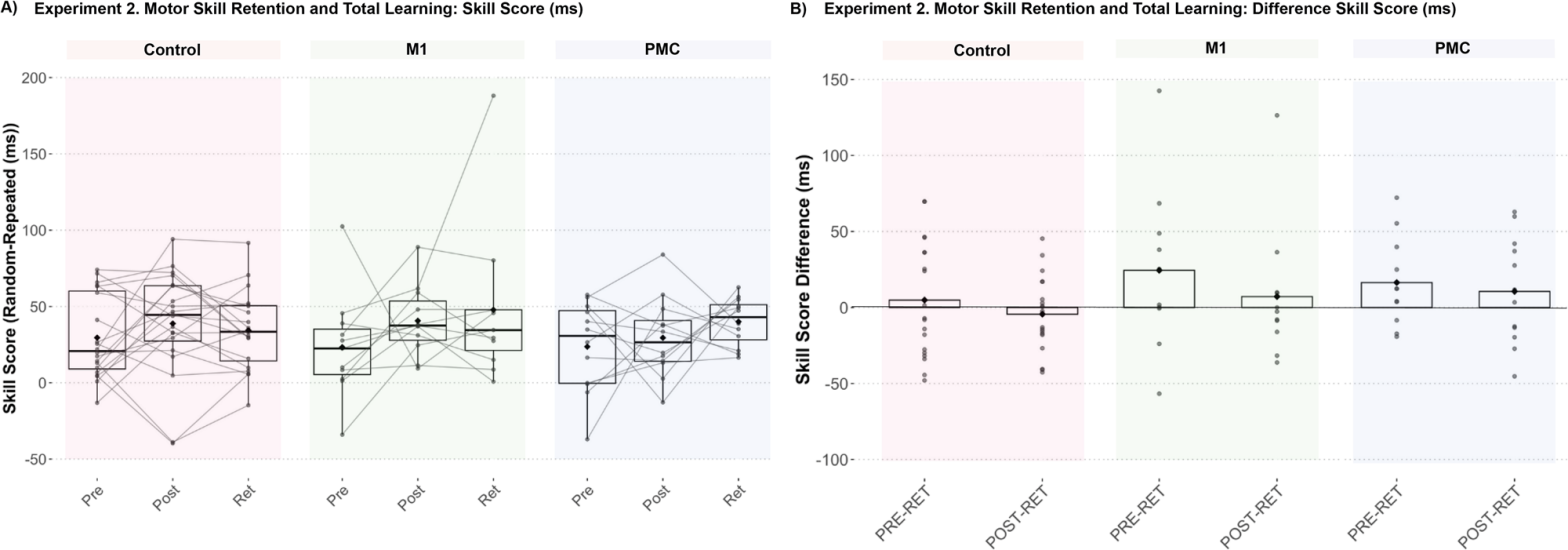
All groups, irrespective of stimulation condition, demonstrated implicit sequence-specific motor skill total learning from pretraining to retention (* *p* < 0.05), but not acquisition or retention.

Independent of the experimental group, mean SS was significantly greater at RET compared with PRE (estimate = −15.23 ms; SE = 5.89; *t* = −2.58; *p* = 0.035; 95% CI [−29.31, −1.15]), but not from PRE to POST (estimate = −10.79 ms; SE = 5.89; *t* = −1.83; *p* = 0.21; 95% CI [−24.86, 3.29]) nor POST to RET (estimate = −4.44 ms; SE = 5.89; *t* = −0.75; *p* = 1; 95% CI [−18.52, 9.63]), and changes in SS were not specific to group (Test × Group interaction, all *p* > 0.1; Fig. 4). While offline, post-training changes in SS (POST to RET) did not reach statistical significance, descriptive statistics suggest that groups either demonstrated minimal sequence-specific skill loss (Control, mean ΔSS = 4.41 ms; SE = 8.35) or enhancement of skill (M1, mean ΔSS = −7.17 ms; SE = 11.26; PMC, mean ΔSS = −10.57; SE = 10.78).

**Figure 5.**
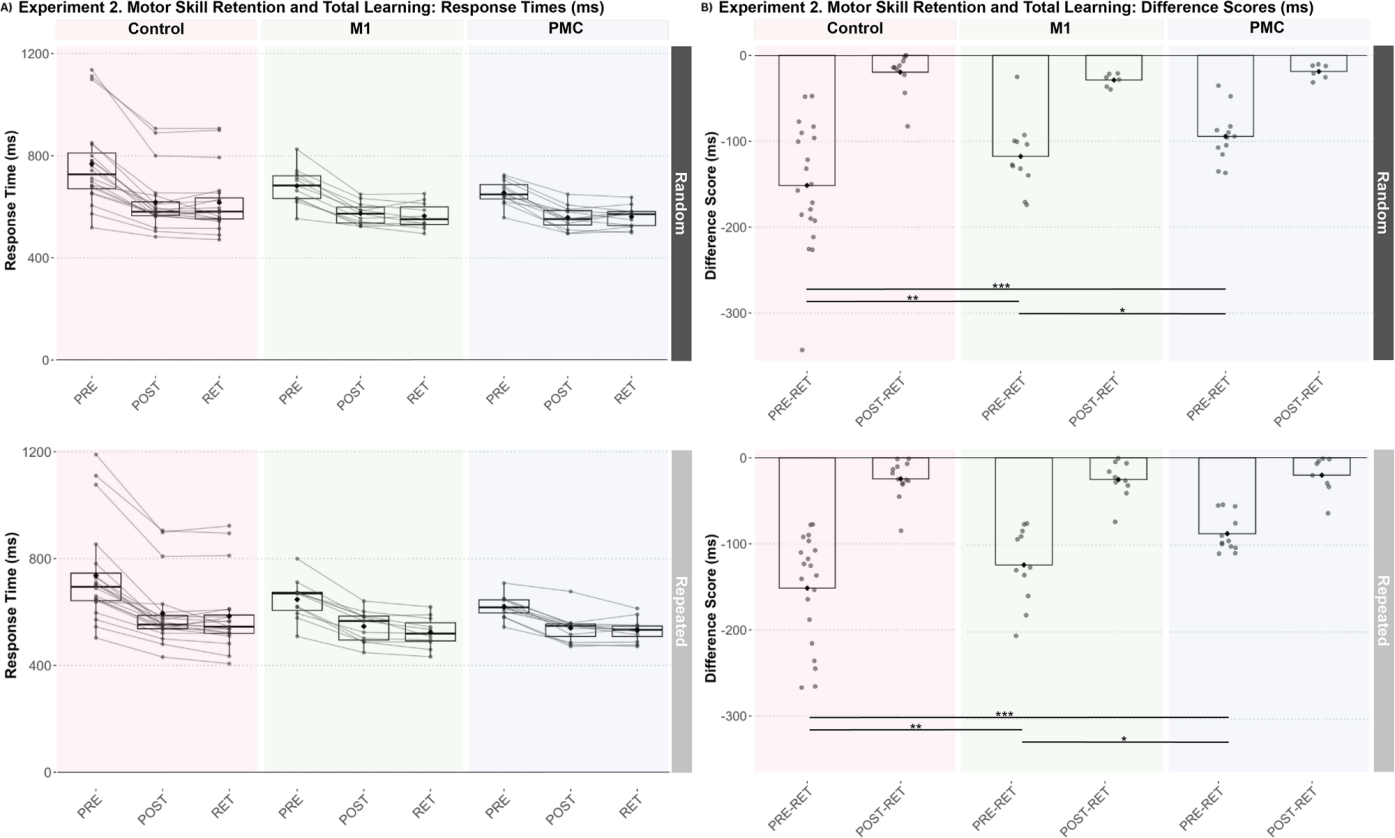
Stimulation groups demonstrated similar retention with less total learning that was not sequence-specific. ***A***, All groups demonstrated decreased response times from pretraining to retention for both random and repeated segments within each test block (***p < 0.001). ***B***, However, less decrease in response times was shown in both stimulation groups compared with controls (**p < 0.01 and ***p < 0.001) with the PMC group demonstrating less decrease in response times compared with the M1 group (*p < 0.05).

Irrespective of the group and test, individuals showed significantly faster response times on sequenced trials compared with random trials (Main Effect of Trial Type, estimate = 30.09 ms; SE = 4.14; *t* = 7.27; *p* < 0.001; 95% CI [21.93, 38.25]) but was not specific to exposure (Trial Type × Test interaction, all *p* > 0.05) nor group (all *p* > 0.05). Similar to Experiment 1, the control group demonstrated a significantly greater reduction in response times than both stimulation groups from pre- to post-training that was not trial-type specific (Group × Test interaction, M1 estimate = 41.51 ms; SE = 12.05; *t* = 3.44; *p* < 0.001; 95% CI [17.75, 65.27]; PMC estimate = 57.86 ms; SE = 11.72; *t* = 4.94; *p* < 0.001; 95% CI [34.75, 80.97]). The control group also showed a significantly greater reduction in response times compared with both stimulation groups from PRE to RET (Group × Test interaction, M1 estimate = 31.14 ms; SE = 12.05; *t* = 2.58; *p* = 0.01; 95% CI [7.38, 54.90]; PMC estimate = 60.76 ms; SE = 11.72; *t* = 5.18; *p* < 0.001; 95% CI [37.65, 83.87]) but not POST to RET (Group × Test interaction, M1 estimate = −10.37 ms; SE = 12.05; *t* = −0.86; *p* = 0.39; 95% CI [−34.13, 13.39]; PMC estimate = 2.90 ms; SE = 11.72; *t* = 0.25; *p* = 0.81; 95% CI [−20.21, 26.01]; Fig. 5). Finally, the PMC group demonstrated significantly less reduction in response time compared with the M1 group from PRE to RET (estimate = −29.63 ms; SE = 13.40; *t* = −2.21; *p* = 0.03; 95% CI [−56.05, −3.20]). When removing the three participants in the control group with prolonged response times at the pretraining test block, there was no longer a significant difference in change in response time from PRE to RET between controls and the M1 group (estimate = 13.88 ms; SE = 10.5; *t* = 1.32; *p* = 0.19; 95% CI [−6.87, 34.63]); however, significantly greater response time reduction from PRE to RET was still observed in controls compared with the PMC group (estimate = 43.50 ms; SE = 10.2; *t* = 4.25; *p* < 0.001; 95% CI [23.29, 63.72]).

### SRTT performance accuracy

Across both experiments, accuracy rates were uniformly high across all groups, time points, and trial types (range, 96–99% correct) in line with prior work (Willingham and Goedert, 1999; Cohen et al., 2005; Table 3).

**Table 3.** Accuracy by group and test

	% accuracy in random trials			% accuracy in repeated trials		
	PRE	POST	RETENTION	PRE	POST	RETENTION
M1	98 ± 2	96 ± 4	96 ± 3	98 ± 2	96 ± 3	97 ± 2
PMC	98 ± 2	97 ± 3	96 ± 2	98 ± 2	98 ± 2	96 ± 3
Control	98 ± 2	96 ± 3	96 ± 5	99 ± 1	97 ± 3	97 ± 3

Mean accuracy (% correct ± SD) for repeated and random trials across experimental groups (M1, PMC, Control) and tests (PRE, POST, RETENTION). All groups demonstrated uniformly high accuracy (96–99% correct responses per trial type within each test block) across trial types and time points suggesting accuracy was prioritized in line with results from prior studies using similar task paradigms (Willingham and Goedert, 1999; Cohen et al., 2005).

## Discussion

The present study investigated the necessary contributions of the PMC and M1 to the acquisition and retention of a sequential motor skill by using TMS to interfere with local activity during a single skill training session. In doing so, all groups demonstrated significant sequence-specific learning shown in both experiments in line with prior work using similar versions of the task. However, we did not observe a significant effect of TMS-based interference on this learning for either active stimulation group, although there was a tendency for the PMC stimulation group to have less sequence-specific learning concordant with our initial hypothesis. Furthermore, disruption of PMC activity had a greater effect on reducing visuomotor skill learning than M1 disruption supporting a potentially distinct role of the PMC in the early stages of skill learning.

### Sequence-specific motor skill learning occurs despite motor cortex disruption

By embedding a repeating sequence within the SRTT, we demonstrated both implicit sequence and general visuomotor skill learning following a single training session replicating prior work (Cohen et al., 2005; Clos et al., 2018). Across groups, significant sequence-specific skill acquisition was demonstrated but was not significantly impacted by TMS-based interference to either the PMC or M1 (Fig. 2). Qualitatively, we observed a lower magnitude of sequence-specific skill acquisition relative to control following PMC stimulation in line with the a priori hypothesis that PMC is involved with sequence formation. These results suggest that PMC may be involved in sequence-specific acquisition, but it may be the case that the dose of TMS interference in the present experiment was insufficient to adequately disrupt training-related sequence learning given only a single TMS pulse was delivered per sequence practiced. It could also be that the dose of training was suboptimal for robust sequence acquisition. Although significant sequence acquisition was demonstrated, the relative magnitude of improvement was modest in all groups. Thus, it will be important to consider the dosage of both neuromodulation and training delivered when characterizing the necessary roles of brain regions to sequence learning in future studies.

It is known that skill acquisition does not necessarily result in skill learning and that processes reflective of transient performance improvements may differ from those supporting durable changes in performance reflective of learning (Kantak et al., 2012; Krakauer et al., 2019). Therefore, to investigate the persistence of skill improvement and the impact of disrupting cortical activity during training, a second experiment employed retention testing the day following training to assess offline skill retention and total learning in a subset of participants. Sequence-specific skill learning was evidenced by higher performance at retention prior to training across experimental groups and no difference in performance at retention compared with post-training suggesting that training-related improvements in sequence skill remained relatively stable during the 24 h offline consolidation period. Taken together, these results suggest that early-stage sequence–specific skill learning does not require undisrupted, normal cortical activity in the PMC nor M1 during training; however, dosing and timing considerations require addressing to further characterize the mechanisms of implicit sequence skill learning.

### Disrupting cortical activity during training reduces general visuomotor skill learning

After characterizing the effects of cortical activity disruption on implicit sequence learning, we then assessed the effects on general visuomotor skill learning by evaluating the change in performance for both random and repeated trial types between test blocks. Both stimulation groups showed impaired motor skill acquisition and total learning irrespective of stimulation site. The disruption of motor skill acquisition in the M1 group further supports the view that M1 is necessary for motor execution and refinement of motor commands with skill training. Prior work has shown that M1 contributes to offline skill gains and stabilization of motor routines (Karni et al., 1995; Doyon et al., 2003). Intriguingly, total visuomotor skill learning was reduced to a significantly greater extent by interfering with PMC activity during training potentially highlighting a distinct role for this region in visuomotor skill learning beyond action selection. This observation may initially seem inconsistent with earlier work showing TMS over PMC delayed responses during a choice reaction time task but did not affect performance on a simple reaction time task (Schluter et al., 1998). However, these prior findings highlighted the role of PMC in resolving competing motor plans rather than executing individual actions. A potential explanation for the effects of PMC disruption on skill acquisition and learning is that the region is important for structuring and chunking motor actions, particularly during the early stages of learning (Hikosaka et al., 2002). Furthermore, prior work showed that PMC activity precedes M1 activation and may be required to establish the timing and coordination of complex movements (Tanji and Shima, 1994). In fact, the sustained PMC activation observed in fMRI studies while performing a SRTT (Honda et al., 1998) is consistent with its hypothesized role in integrating sensory information and contextual cues to scaffold emerging motor routines.

While the motor task was structured as a simple button press in response to a discrete visual cue, the randomized color-location mappings introduced perceptual ambiguity. This ambiguity added a level of stimulus–response uncertainty, effectively transforming the task into one with increased action selection demands. Under such cognitively demanding conditions, the PMC may have an increased role in integrating perceptual input and selecting the appropriate response that could explain, in part, the general reduction in motor skill learning when PMC activity was disrupted during training compared with M1 and controls.

### Considerations of baseline differences in skill performance and protocol design

An additional consideration for interpreting the current results lies in potential baseline variability across participants and stimulation groups. While group assignment was randomized, differences in baseline motor performance or individual responsiveness to TMS may have introduced variance that obscured more subtle effects on implicit sequence and/or general visuomotor skill learning. Additionally, although EMG onset was monitored to ensure appropriate timing of TMS, trial-by-trial variability in TMS delivery relative to planning and initiation may have impacted the magnitude of activity disruption (Steel et al., 2016). However, the overall behavioral effects of TMS disruption of PMC and M1 activity relative to the sham TMS control group remained largely unchanged when three individuals in the control group that demonstrated distinctly slower response times during pretraining assessments were removed from the analyses suggesting baseline differences were not driving the observed effects of TMS disruption.

### Implications and future directions

Our findings demonstrate common and distinct contributions of the PMC and M1 during early motor skill learning. While M1 is thought to be more important for the execution and consolidation of movement routines, the PMC appears to facilitate broader planning and task adaptation, particularly under implicit learning constraints. The inclusion of a well-characterized sham TMS group replicated prior findings (King et al., 2022) and inclusion of retention testing in the second experiment supports the conclusion that interfering with PMC and M1 during motor skill training reflects disruption of skill acquisition rather than being attributable to general attentional or fatigue-related effects.

To further probe the causal contributions of these and associated cortical motor areas, future studies should employ repetitive or paired-pulse stimulation protocols, integrate electroencephalography or fMRI for online validation of disruption, and/or adopt adaptive stimulation strategies that precisely align cortical activity disruption to discrete neural events that contribute to movement generation. Shorter sequences and embedded repetition structures may also enhance statistical learning signals and reduce task difficulty, improving sensitivity to group differences.

It is also worth noting that we employed a posterior-to-anterior (PA) current direction for TMS delivery, which preferentially activates transsynaptic inputs to pyramidal projections (early I-waves). However, an anterior-to-posterior (AP) current direction has been shown to preferentially recruit polysynaptic projections (late I-waves), which may reflect more complex circuitry including projections from PMC to M1 (Mirdamadi et al., 2017; Hannah et al., 2018; Spampinato et al., 2020; Neva et al., 2021). Given that motor corticospinal output evoked by AP current can be selectively modulated by attention and task demands, future studies could employ AP-induced current to more directly probe PMC's functional contributions to implicit sequence-specific motor skill learning. Comparing the effects of current directions could elucidate whether observed behavioral results reflect primarily M1-dependent processes or may be influence to an extent by premotor–motor interactions.

## Conclusion

Our primary study findings suggest the necessary role of both the PMC and M1 in the early acquisition of a novel visuomotor skill, rather than implicit sequence skill, with a distinct role of the PMC in overall motor skill learning. Given the importance of motor skill learning to enhancing and regaining function, determining the necessary mechanisms for learning can be exploited in the future by tailoring training structures to individual cortical activity biomarkers and/or developing precision neuromodulation strategies that selectively enhance neural activity supporting motor skill learning and recovery of function.

## Synthesis

Reviewing Editor: Alexander Soutschek, Ludwig-Maximilians-Universitat Munchen Decisions are customarily a result of the Reviewing Editor and the peer reviewers coming together and discussing their recommendations until a consensus is reached. When revisions are invited, a fact-based synthesis statement explaining their decision and outlining what is needed to prepare a revision will be listed below. The following reviewer(s) agreed to reveal their identity: danny Spampinato.

The reviewers agreed that the revision substantially improved the quality of the manuscript. In particulat, the added skill score analysis, improved reporting of TMS timing and exclusions, and clearer figures substantially strengthen the manuscript. The revised interpretation - that stimulation primarily affects general motor performance rather than sequence-specific learning - is appropriate and improves conceptual clarity.

The reviewers make the following suggestions to further improve the manuscript: Ensure that the title, abstract, and discussion consistently reflect the updated interpretation and avoid overstating selective effects on sequence learning. Please clarify whether the composite skill score analysis was pre-planned or added during revision. Specify the analysis software used and consider adding a brief quantitative comparison to confirm that excluded participants did not bias results.
